# Lithospheric delamination as the driving mechanism of intermediate-depth seismicity in the Bucaramanga Nest, Colombia

**DOI:** 10.1038/s41598-023-50159-4

**Published:** 2023-12-27

**Authors:** Diego Pérez-Forero, Ivan Koulakov, Carlos A. Vargas, Taras Gerya, Nassir Al Arifi

**Affiliations:** 1 Trofimuk Institute of Petroleum Geology and Geophysics, SB RAS, Prospekt Koptyuga, 3, Novosibirsk, Russia 630090; 2 Novosibirsk State University, Pirogova 2, Novosibirsk, Russia 630090; 3 Skolkovo Institute of Science and Technology (Skoltech), Bolshoy Blrd 30/1, Moscow, Russia 121205; 4 Institute of the Earth’s Crust SB RAS, Irkutsk, Russia; 5 Department of Geosciences, Universidad Nacional de Colombia at Bogotá, Bogotá, Colombia; 6 Department of Earth Sciences, ETH Zurich, Sonneggstrasse 5, 8092 Zurich, Switzerland; 7 Chair of Natural Hazards and Mineral Resources, Geology and Geophysics Department, King Saud University, P.O. Box 2455, 11451 Riyadh, Saudi Arabia

**Keywords:** Geodynamics, Seismology

## Abstract

The Bucaramanga nest (BN) is an area of exceptionally strong intermediate-depth seismicity localized in a narrow zone at 150–170 km depth beneath the continental plate in Colombia. To explain the very unusual mantle seismicity cluster in this area, we built a seismic velocity model in the vicinity of BN with the use of local earthquake tomography and developed a numerical hydromechanical model. Our seismic model shows a strong high-velocity anomaly at 130–167 km coinciding with the BN seismicity. The relocated seismicity can be separated in two clusters. We propose that the upper BN cluster at ~ 130 km depth is attributed to dehydration embrittlement, whereas the lower BN cluster at ~ 150 km depth coinciding with the high-velocity body is caused by lithospheric delamination, creating a “drip” that falls down over the subducting oceanic plate, enhancing fluid release from the slab, potentially increasing seismicity.

## Introduction

A particular kind of intermediate-depth earthquake concentration or clustering beneath continents, known as an earthquake nest located in the mantle, is characterized by a high activity rate that is isolated from nearby activity^1–3^. There are few areas in the world where this phenomenon occurs, of which some notorious examples are Vrancea (Romania)^4^, Pamir-Hindu Kush (Afghanistan)^5,6^, and Bucaramanga seismic nest (BN) in Colombia.

**Figure 1:**
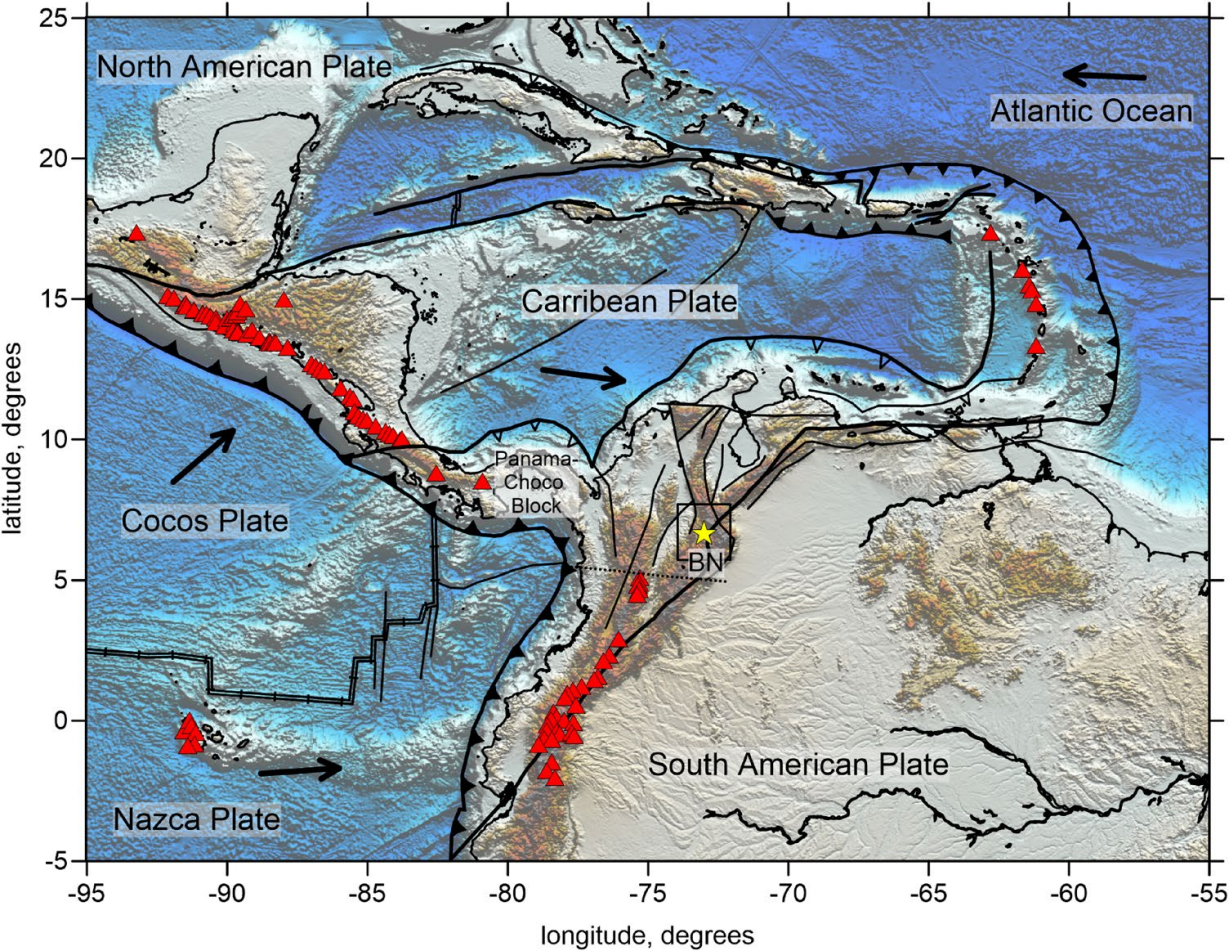
Main tectonic features in NWSA and the Caribbean. The red triangles indicate volcanoes; the square highlights the study area; the dashed line depicts Caldas Tear; the yellow star indicates BN.

The BN is a particularly strong seismicity cluster located in northwestern South America (NWSA), in the Colombian Andes (Fig. 1), which produces approximately 60% of the total earthquakes recorded by National Seismological Network of Colombia (NSNC). The BN corresponds to a seismically active zone at depths between 150 and 170 km having a volume of approximately 800 cubic km^3,7,8^. This nest is particularly interesting because in contrast to Vrancea and Pamir-Hindu Kush, the BN is more restricted in volume, and it is considered as the densest concentration of intermediate-depth earthquakes in the world^2^.

The structure beneath NWSA, has been previously studied by several authors aiming to answer two main conundrums: the relationships between oceanic and continental plates below NWSA, and the physical mechanisms that generate the seismic activity in the BN. However, the results of seismic tomography studies produced by different authors and algorithms are not fully consistent and in some cases give contradictory conclusions. A brief overview of the previous results and the existing interpretations are given in the next section with geological settings.

In this study, we mostly focus on studying the detailed seismic velocity structure of the BN itself, using the LOTOS seismic tomography code^9^. Here we pay special attention to the verification of the distinct seismic velocity patterns in the vicinity of the BN. Based on these deep structure configurations, we propose a new mechanism causing the BN seismic activity. To prove that this mechanism is physically plausible, we develop a numerical hydromechanical model simulating the processes associated with the BN.

## Tectonic setting

As shown in Fig. 1, the Caribbean and NWSA are the very complex regions with several subduction zones oriented in different directions. The tectonic processes in this zone are mostly controlled by the subduction of oceanic Nazca, Cocos and Caribbean Plates underneath the continental South American plate. The interaction of these plates causes active tectonic deformations in the crust and complex processes in the mantle leading to active seismicity at various depth levels^7,8,10,11^.

In the present time, the Caribbean plate obliquely underthrusts NWSA at a rate of ∼20 mm/y eastward, with a very small component directed southward. To the east, off the Venezuelan coast, the plate convergent regime transfers to the strike-slip^8^. However, some authors suggested that in recent geological past, the orientation of subduction of the Caribbean Plate was different and provided slab dipping underneath NWSA^10,12^.

The Nazca plate, which is the part of the Pacific Ocean, currently moves eastward at a rate of ∼54 mm/y almost perpendicularly relative to the coast of South America^10,13^. The evolution of the volcanic arc over the past 14 Ma along Colombia’s Pacific margin^14^ indicates that the volcanic activity in this area corresponds to only the Nazca slab. Lack of volcanism to the north of 5°N coincides with flattening of the slab, a process that started ~ 10 Ma due to subduction of thicker crust of the Panama indenter^11^. The boundary between the steep and flat segments of the Nazca slab at 5°N corresponds to the Caldas tear zone.

Several different models have been proposed to determine the configuration of the Caribbean and Nazca Plates below NWSA. Pennington^15^ argued that subducted parts of the Panama block and Pacific Ocean are present below NWSA as three slab segments. In this concept, Bucaramanga nest belongs to the subducting Caribbean Plate. Taboada et al.^8^ identified the northward low-angle subduction of the paleo-Caribbean plateau, which overlaps with the steep Nazca slab. The tomography study by van der Hilst and Mann^16^ has also identified two slab segments corresponding to the paleo-Caribbean and Nazca Plates, but suggested that BN belongs to the Nazca segment. Based on another tomography study, Chiarabba et al.^17^ argued that both flat and steep segments below NWSA belong to the same Nazca Plate that is teared at 5° N. In this interpretation, BN corresponds to the location where the slab turns from the flat to the steep shape. Similar conclusions were made by Vargas and Mann^11^ based on the results of the coda-wave attenuation tomography. Syracuse et al.^18^ performed combined inversion of the body wave and surface wave data together with the gravity field and clearly imaged the presence of the Nazca slab tear at 5° N. However, they proposed that the flat part of the slab is composed of two segments corresponding to the Nazca and Caribbean plates. In this concept, the BN seismicity occurs at a boundary between these segments. Based on another seismic tomography model, Londoño et al.^19^ identified both the Caribbear and Nazca slabs and proposed that BN is located at the contact between them, Another mechanism was proposed by Cornthwaite et al.^20^ based on the results of finite-frequency teleseismic tomography, who identified three separate segments of the Caribbean Plate subducting southward, one of which is completely detached. Although the Bucaramanga zone was located outside the resolved area of this study, the authors speculated that the BN might be associated with the detached segment of the Caribbean Plate. Based on further interpretation of this teleseismic model, Sun et al.^12^ revealed a large overlapping of the Caribbean and Nazca Plates for more than 200 km and concluded that the BN is associated with the Caribbean Plate. The receiver function study by Mojica Boada^21^ has identified three segments of the Nazca Plate and two segments of the Caribbean Plate. They suggested that BN was associated with the interaction of the two northernmost slab segments.

Regarding the origin of BN, several authors have investigated the mechanisms responsible for the extremely strong seismic activity in the mantle from physical and geodynamical perspectives. However, their conclusions often appear to be not consistent and even contradictory. First, as was discussed above, there is no consensus about the association of BN to the subducting plates in this area. Second, different seismic tomography models imaged the area of BN differently. For example, the model of Chiarabba et al.^17^ exhibits low Vp, low Vs and low Vp/Vs ratio in the area of the BN cluster. Syracuse et al.^18^ reported low Vp, high Vs and high Vp/Vs ratio. High velocities for the both P and S waves were obtained in Ref.^19,22^. All these examples show that the problem of the origin of the BN seismicity is far from the definitive solution. Therefore, in this study, we revisit generally the same data as used in some of the previous studies, and focus on extracting the maximum relevant information on the BN and proving the reliability of the derived structures.

As shown in this overview, the information on the shapes of plates below NWSA and Caribbean basin presented by different authors is not always consistent and often contradictory. In this study, we mostly focus on understanding the origin of the BN seismicity, but do not pretend to provide any argument related to the slab geometries and names. Although we find it more plausible that the BN is associated with the Nazca Plate, we cannot exclude an alternative configuration with the Caribbean Slab. Therefore, throughout the paper, we avoid any explicit naming of the slab, with which the BN is associated.

## Seismic tomography inversion

**Figure 2:**
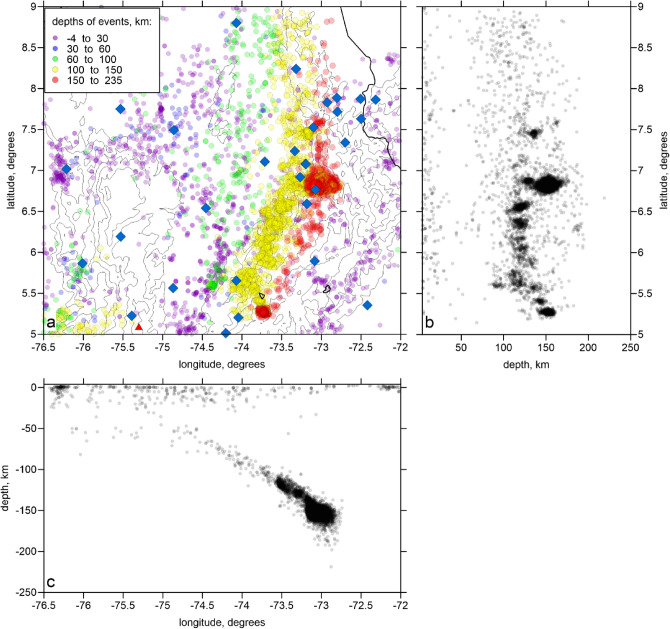
Distributions of data used for tomographic inversion. (**a**) Map view of the stations (blue diamonds) and earthquakes colored according to their depths. Contour lines indicate topography with the interval of 1000 m. (**b**,**c**) are the projections of the events on two orthogonal planes.

Seismic data was provided by the Colombian Geological Survey (SGC) and comprised the arrival times of *P* and *S* waves from regional events registered by 231 seismological stations that occurred from 1993 to 2012 (Fig. 2). For tomography, we selected the data according to the following criteria: (1) both stations and events should be located within the radius of 200 km centered in BN; (2) the number of the *P* and *S* picks should be larger or equal 17; (3) the residuals after the preliminary source locations in the 1D model should be less than 1.5 s. As a result of selection, we obtained the dataset with 22,100 events, 289,275 *P*-rays and 258,327 *S*-rays, which corresponded to 24.7 picks per event on average. These events are presented in Fig. 2 in map view and in vertical projections.

**Figure 3:**
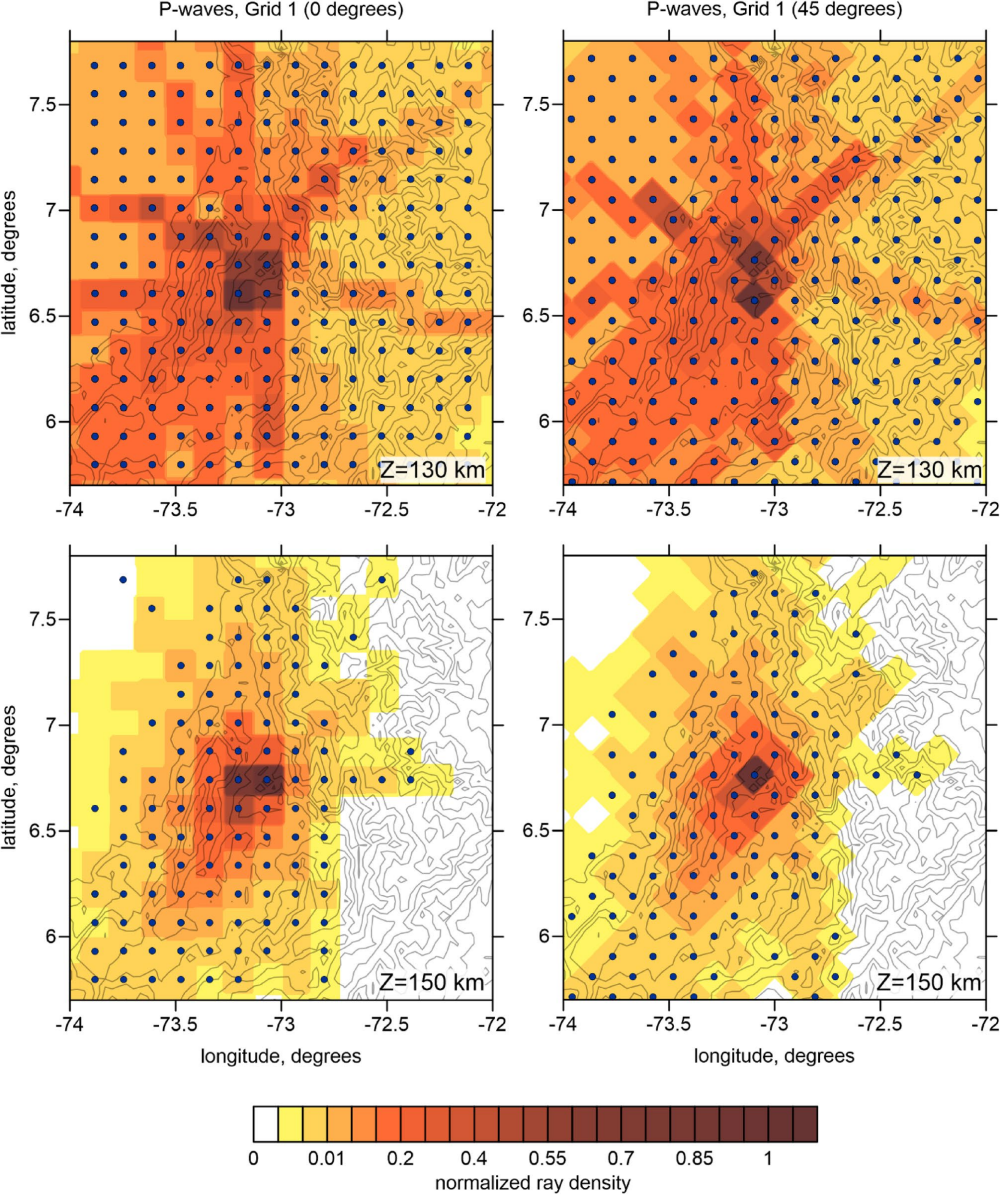
Distributions of nodes of two parameterization grids (blue dots) with different basic orientations used for the inversions. Background is the normalized ray density in two depth levels. Contour lines indicate topography with the interval of 1000 m.

A 3D velocity tomography inversion was performed using the LOTOS code^9^, which has been used to explore the structure beneath some areas with similar geological settings, such as the Central Andes^23^, Hokkaido^24^, Pamir-Hindu Kush^25^ and the Vrancea seismic nest^26^. More detailed description of the LOTOS code workflow and some controlling parameters are described in Method Section. To reduce the parameterization effect to the result, we performed inversions in four grids having different basic orientations (as shown in Fig. 3) and then averaged the results in one 3D model.

The main result of the inversion is a tomography model including the 3D distributions of *P* and *S* wave velocity (*Vp, Vs*) anomalies, as well as the locations of seismic events. The calculation for the *Vp/Vs* ratio appeared to be not robust in this case, as it was strongly dependent on the relationships between the damping values for the *Vp* and *Vs* models; therefore, this parameter was not used for interpretation.

**Figure 4:**
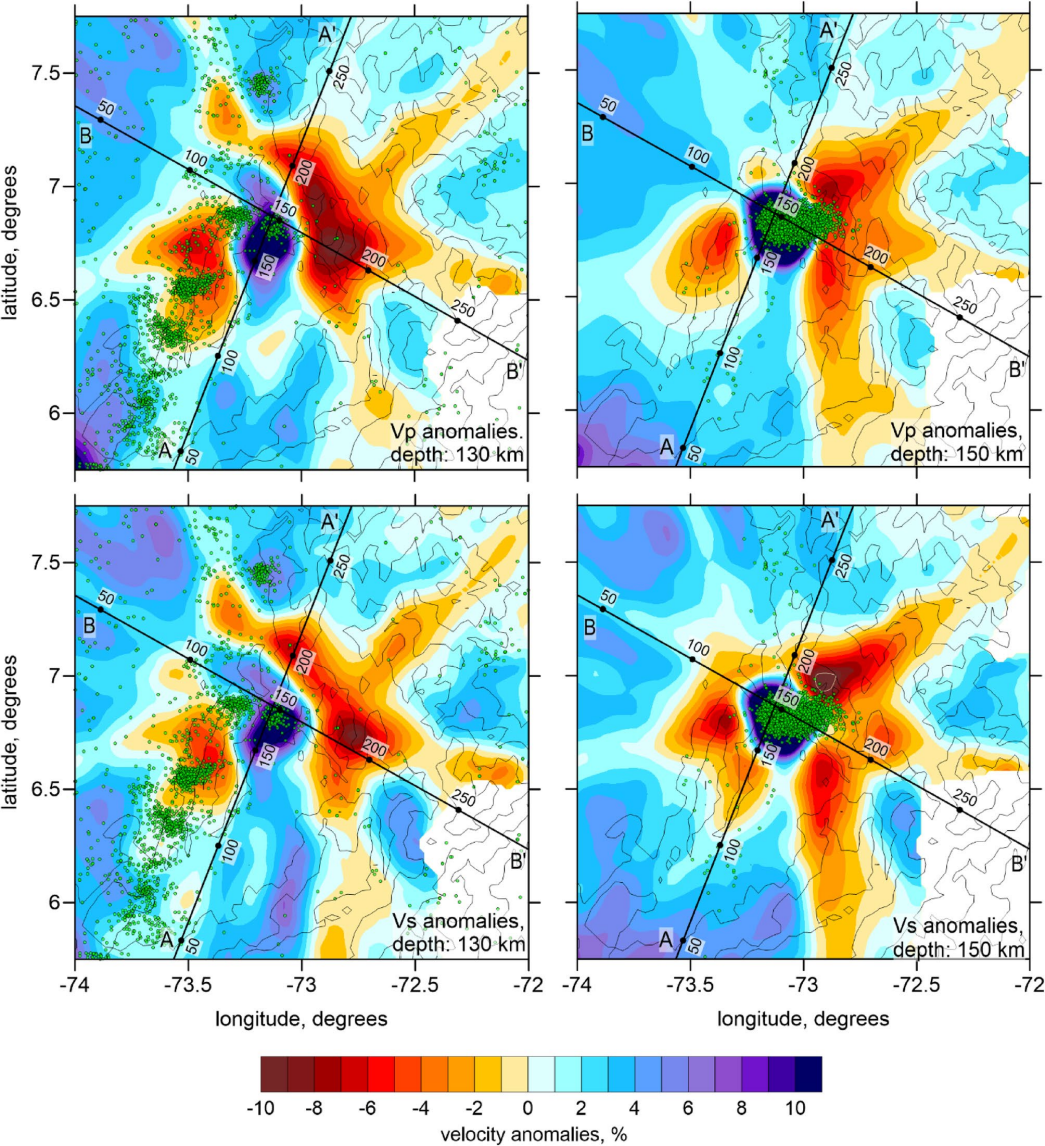
P and S wave velocity anomalies in two horizontal sections. The green dots indicate the locations of seismic events at distances of < 10 km from the sections. In the blank areas, the distance to the nearest node is > 20, meaning that there is no enough data for tomography.

**Figure 5:**
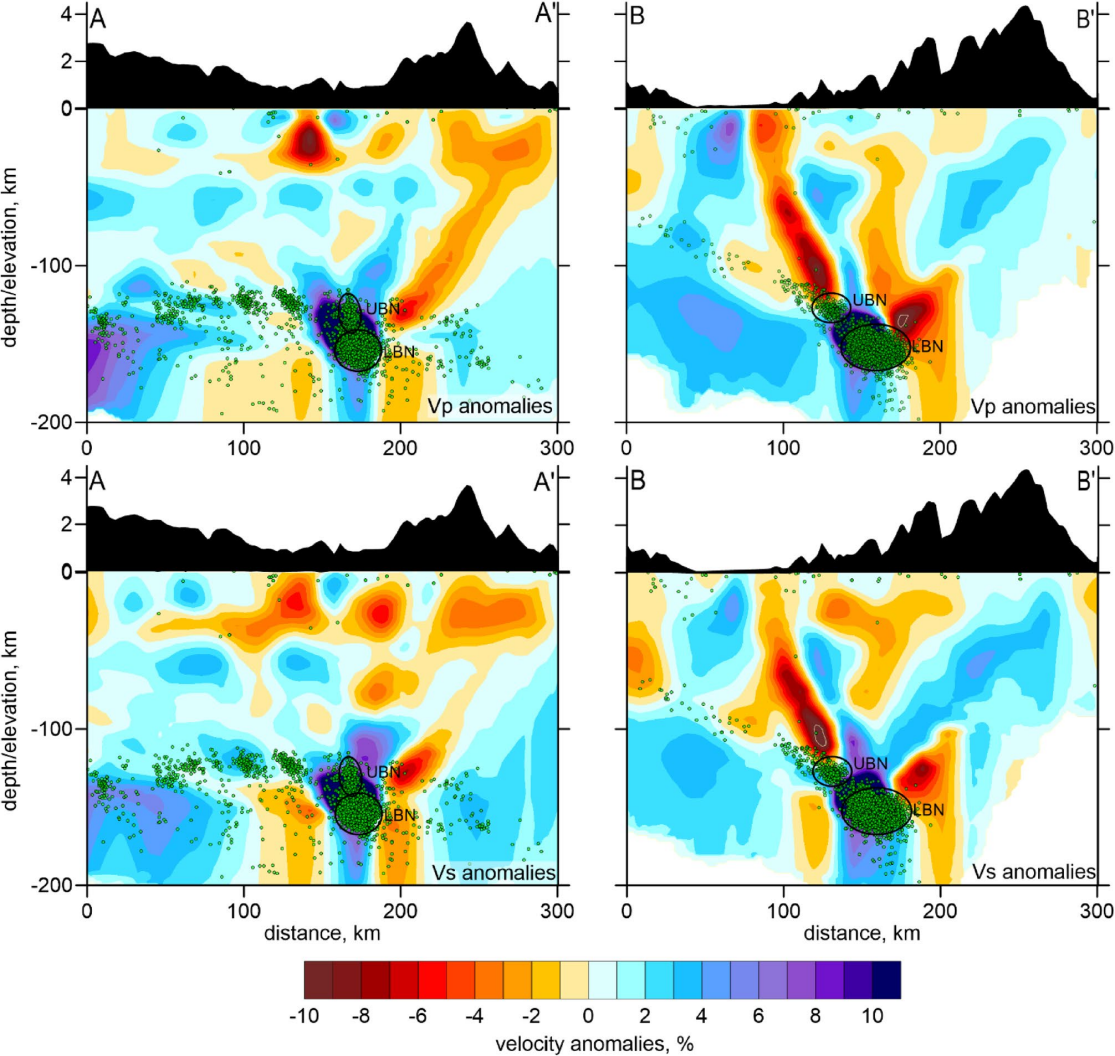
P and S wave velocity anomalies in two vertical sections indicated in Fig. 4. The green dots indicate the locations of seismic events at distances of < 20 km from the sections. The exaggerated relief along the sections is presented above the profiles. In the blank areas, the distance to the nearest node is > 20, meaning that there is no enough data for tomography. The upper and lower BN are indicated by UBN and LBN, respectively, and highlighted by ellipses.

**Figure 6:**
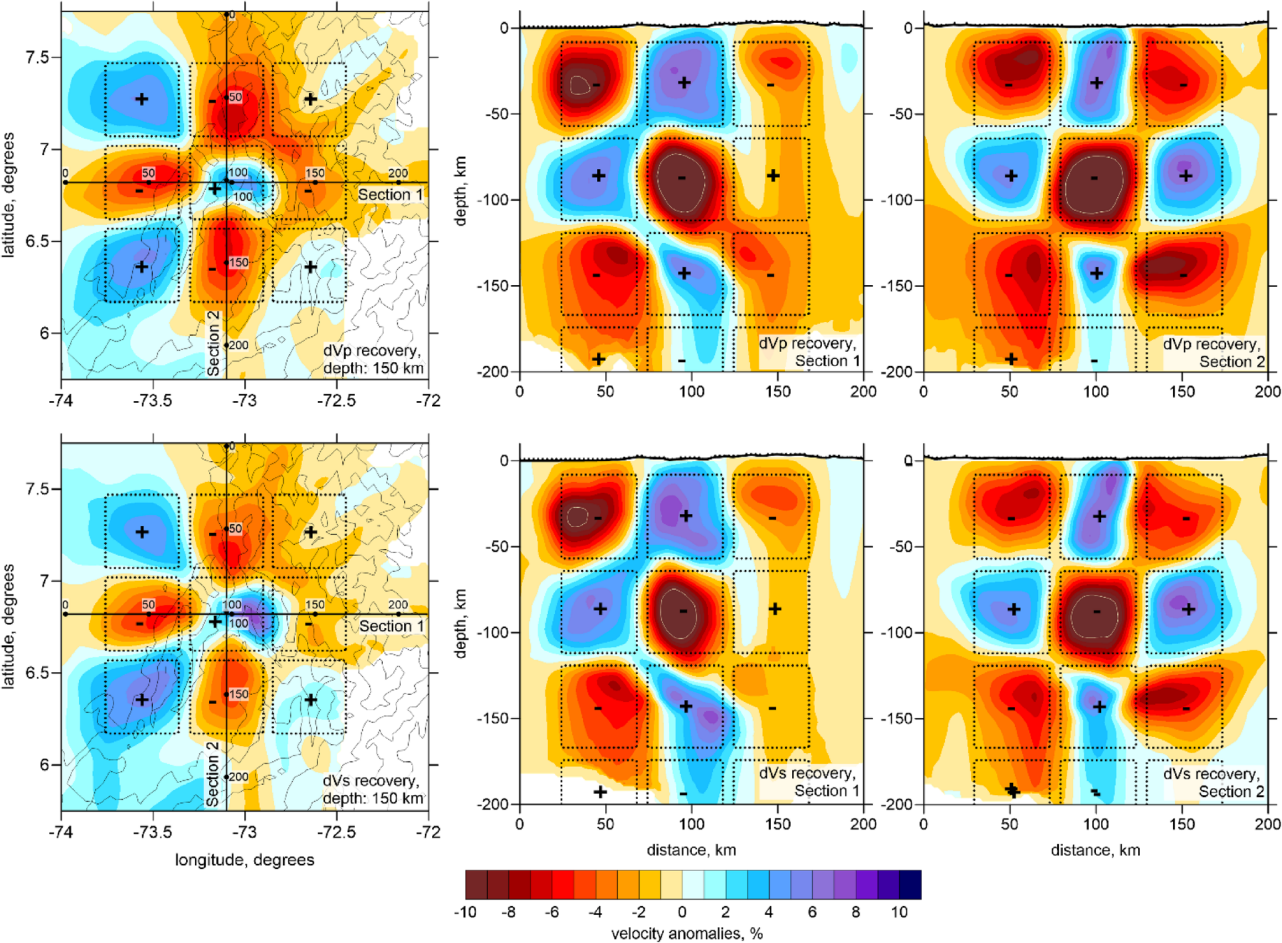
Checkerboard test. The recovered anomalies of Vp and Vs are presented in one horizontal and two vertical sections. The dotted lines indicate the shapes of the synthetic anomalies. The pluses and minuses indicate the polarity of the synthetic anomalies. The contour lines in the maps indicate the topography with the interval of 1000 m.

In Figs. 4 and 5, we present the derived *P* and *S* wave velocity anomalies in two horizontal and two vertical sections with SW-NE and NW–SE orientations together with the distribution of seismicity. The reliability of the derived structures in the BN area was verified using a series of synthetic tests. The methodology of the synthetic testing is presented in the Method Section. Figure 6 presents the checkerboard test with alternated anomalies with the size of 43 × 43 × 48 km separated by spaces with zero anomalies of 7 km width. The amplitude of the anomalies is ± 8%. The recovered anomalies of dVp and dVs are presented at the depth of 150 km and in two vertical sections. We can observe that the anomalies in the lower row of the checkerboard are not correctly recovered due to the trade-off between the velocity and source parameters that smears the deep structures at the lower part of the seismicity cluster. At the same time, the anomalies in the upper three rows are recovered correctly, which demonstrates satisfactory resolution of the model above 150 km depth.

**Figure 7:**
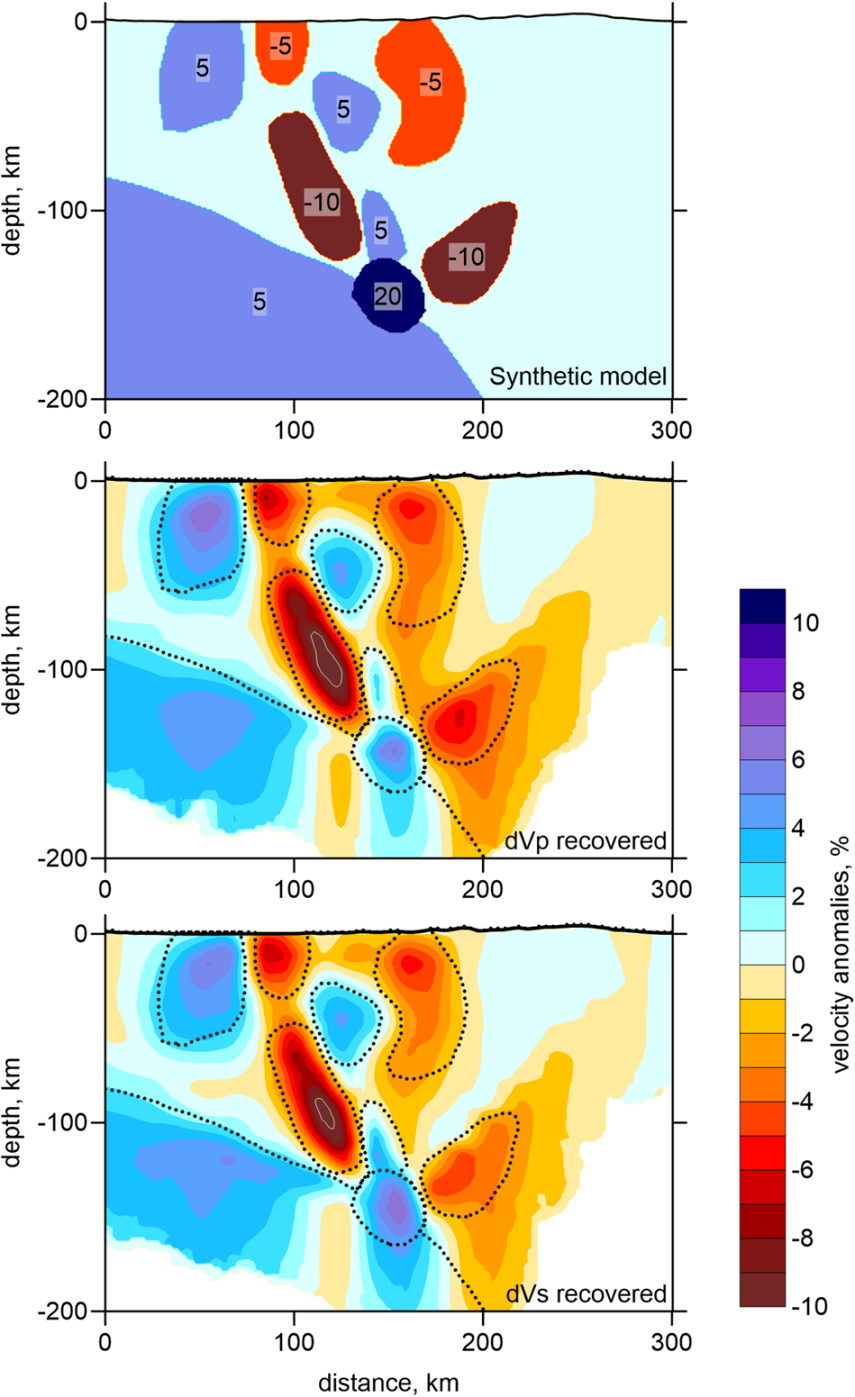
Synthetic test with realistic anomalies defined along section B-B’, same as in Fig. 5. The upper plot presents the initial synthetic model with the indications of anomaly amplitudes, in percent. The middle and lower panel show the recovery results for the P and S velocity anomalies. The dotted lines highlight the shapes of the synthetic anomalies.

In Fig. 7, we present another test, in which we defined free-shaped anomalies along the vertical section B-B’ representing general patterns observed in the main model (Fig. 5). The values of the P and S wave velocity anomalies in the synthetic model, which were identical in this case, are indicated in the upper panel in Fig. 7. The thickness of the anomalies in the direction across the section was 50 km. It can be seen that all anomalies are recovered in correct locations. Some of them are smeared in the vertical direction due to dominant orientation of the ray paths. Important that the slab-related anomaly is correctly recovered, except for the area around the drip, where it is perturbed by spurious low-velocity anomalies. The same patterns were also observed in the main model and were probably caused by the problem of the trade-off between the deep source locations and velocity distributions.

**Figure 8:**
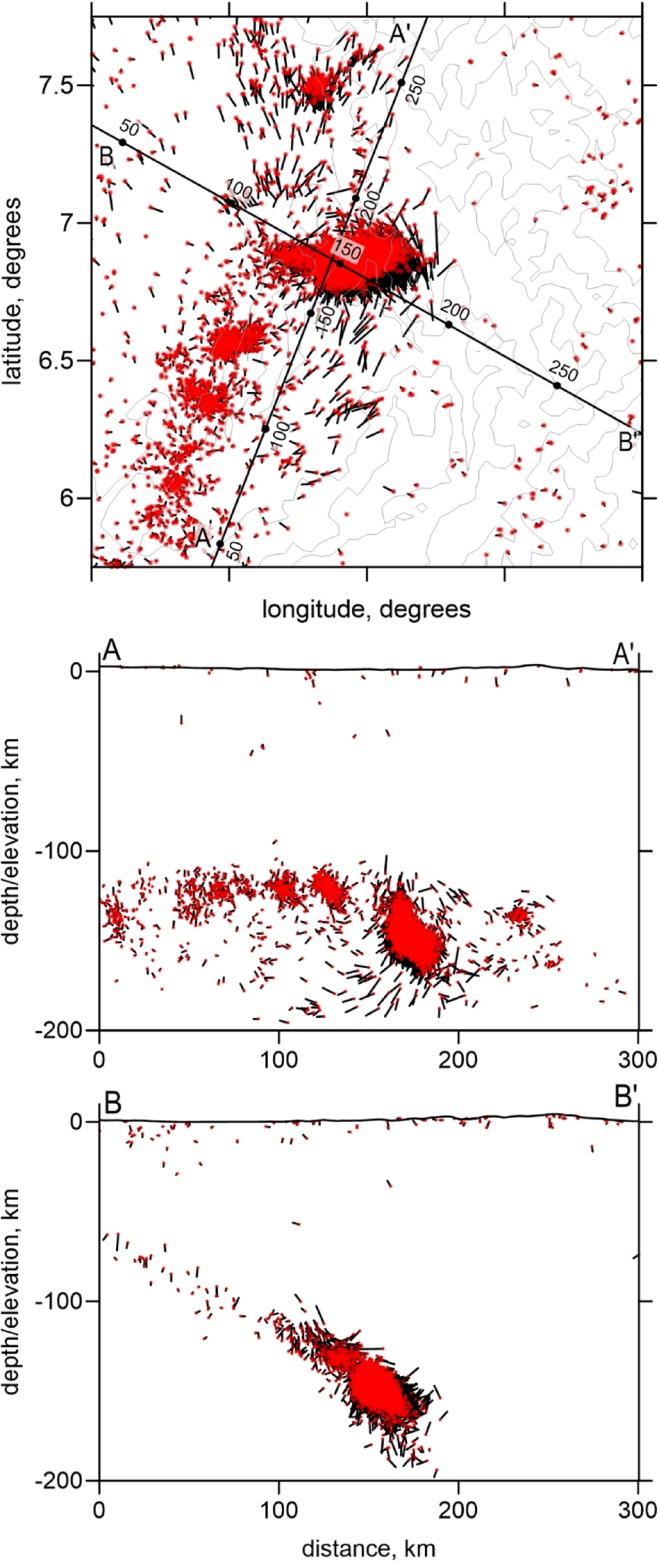
Source mislocations in map view and in the vertical sections after recovery of the synthetic model presented in Fig. 7. Red dots are the derived solutions and the bar ends indicate the true locations. In vertical sections, the events located at distances < 30 km from the sections are shown.

The synthetic testing allows also assessing the accuracy of source locations. In Fig. 8, we present the source location results obtained for the free-shaped synthetic model shown in Fig. 7. The relocated events and misfits with respect to the true locations are presented in map view and in two vertical sections. In this case, the average error of source locations is 4.32 km. Some regular shifts of the events are associated with limited capacity to recover velocity structures in some parts of the study area. Despite all above-mentioned problems, the synthetic tests demonstrate generally satisfactory reliability of the main structures discussed in this study.

The resulting velocity model offers a closer look at the area surrounding the BN compared to all previous tomography studies. The most prominent feature observed in the model (Figs. 4 and 5) is a strong high-velocity anomaly for both *Vp* and *Vs* at depths between 130 and 167 km. The range of occurrence of this high-velocity body roughly coincides with the distribution of intermediate-depth seismicity, which leads to safely assuming this anomaly to be the part of the BN system.

Besides the P and S wave velocity models, our tomography inversion provided the updated solutions for the seismicity locations derived in the resulting 3D model. In this study, we mostly focus on the BN seismicity and on events on the slab. In a horizontal section at a depth of 130 km in Fig. 4, we can observe a belt of slab-related seismicity composed of several small clusters, but forming a nearly linear structure. Note that this seismicity lineament is observed in both sides of the BN, which indicates that the BN is located in the middle part of the subducting plate, and not at the edge, as proposed by some authors^18,19^.

As seen in source distribution, seismic activity within the BN starts at around 130 km and reaches the maximum at the depths of 150–160 km, where the most intensive high-velocity anomaly is observed. Looking closely at the distribution of sources in the BN area, we can distinguish two clusters: the upper one (UBN) separated from the lower one (LBN), which is deeper and larger. We can consider the LBN as the BN per se; while the UBN, which is just outside the positive anomaly, might be related to other processes. Below the depth of 180 km, there are a very few events recorded and, therefore, no velocity information can be retrieved.

The high *Vp* and high *Vs* anomalies associated with the BN observed in our model agree with the previous results^19,22,23,27^. However, they appear to be inconsistent with those obtained by Chiarabba et al.^17^, who showed low *Vp, low Vs* and low *Vp/Vs* ratio, and Syracuse et al.^18^, who found low *Vp,* low *Vs* and high *Vp/Vs* ratio. We agree with Londoño et al.^19^, who argued that this kind of discrepancy could be due to the different data coverage and too coarse grid spacing in the earlier studies. Note also that the previous tomography studies investigated larger areas and had generally lower resolution than in our model, in which we explicitly focused on the BN seismic structure.

## Numerical hydromechanical model

**Figure 9:**
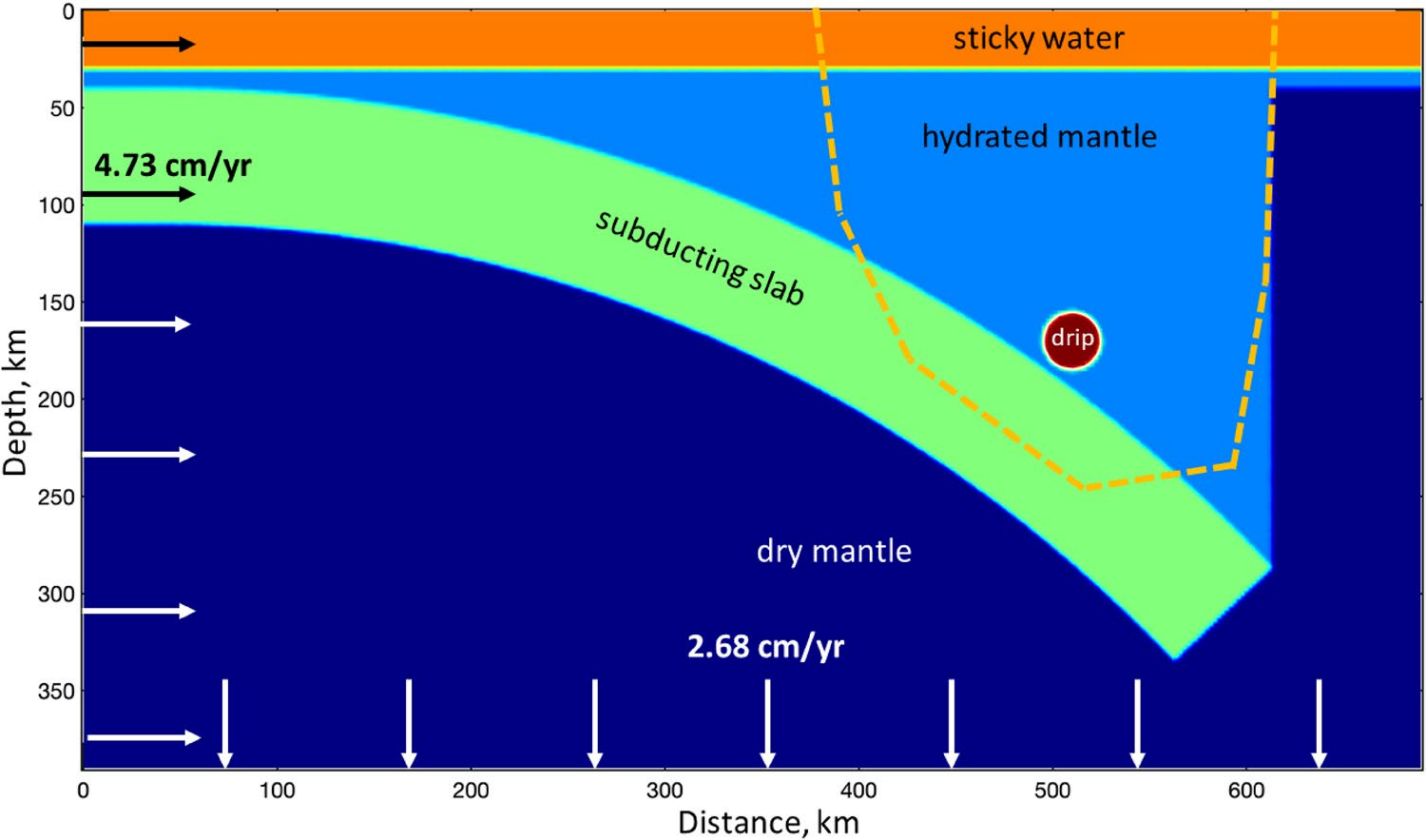
Numerical hydromechanical model geometry and boundary conditions. Arrows and numbers indicate velocity on the boundaries. The orange dashed line indicate an approximate location of the resolved area in the seismic tomography model.

**Figure 10:**
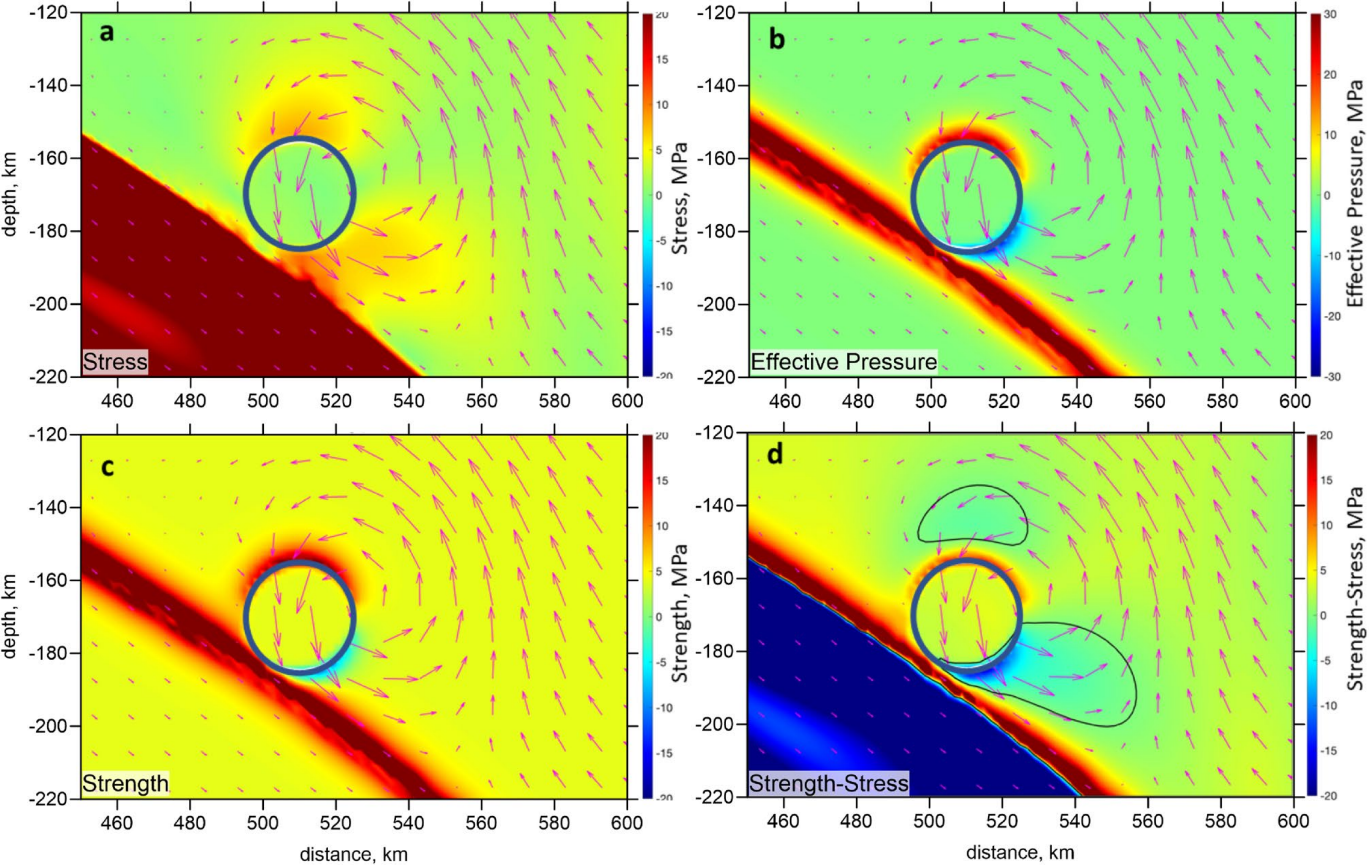
Results of hydromechanical numerical modeling (Methods) of a circular dense eclogitic drip interaction with subducting slab. Colors show different physical parameters with their respective units given on top of each plots: (**a**) deviatoric stress (square root of the second stress invariant is shown), (**b**) effective pressure (the difference between the total and fluid pressure), (**c**) brittle-plastic strength, (**d**) stress-strength difference. Blue contour shows boundary of the drip. Arrows show velocity of the solid matrix. Black contours in d outline two areas of the negative stress-strength difference, which should be thus characterized by elevated seismicity.

In order to test numerically our conceptual model following from the seismic tomography results, we developed a hydromechanical subduction model (Method Section), in which the dense circular eclogitic drip collides with the subducting slab (Fig. 9). The initial parameters of the model correspond to the available configuration and displacement rate of the Nazca Plate^8,13^. The approximate location of our study area is indicated in Fig. 9 by the orange dashed line. To build this model, we used the algorithms developed in Ref.^28^. The purpose of this test was to check if the proposed scenario reconciles with the peculiar distribution of seismicity documented in the region of the BN. Panels a, b, c and d of Fig. 10 show respectively the distribution of deviatoric stress, effective pressure (i.e. the difference between the total and fluid pressure), brittle-plastic strength and stress-strength difference computed with the model. Deviatoric stress (Fig. 10a) shows two clear maxima located both above the drip and at its frontal edge above the slab. In contrast, effective pressure (Fig. 10b) shows maximal values on top of the drip and minimal values at its frontal edge subjected to compressive deformation due to elevated fluid pressure there. As the result, strength of rocks (Fig. 10c) also strongly decreases in front of the drip. The resulting distribution of stress-strength difference (Fig. 10d) shows two distinct minima, where fluid pressure induced seismicity can be expected: one region above the drip and another in front of it (cf. black contours in Fig. 10d). This numerical modeling prediction appears in good agreement with seismicity distribution around the BN (Figs. 2 and 5), thereby supporting our conceptual model that the BN likely represents an eclogitic drip, which should be characterized with high density, relatively low viscosity (compared to the mantle) and low fluid content (porosity) and permeability. According to the numerical model, the observed elevated seismicity of the BN region should mainly be triggered by increased deviatoric stresses and elevated fluid pressure created by the downward propagation and collision of the eclogitic drip with the subducting slab.

## Discussion

Owing to distinct features of seismic velocity and seismicity distributions identified in our tomography results, we can reconsider the causes of abnormally strong mantle seismicity in the BN. In Section B-B’ (Fig. 5), we see a continuous seismicity zone that follow the subducting slab dipping with the angle of ~ 30°. Note that this seismicity lies in a low-velocity zone, which is identified above the high-velocity slab-related anomaly. We propose that this low-velocity anomaly might represent the anomalously thick crust related to the extinct island arc that began subducting below NWSA about 12 Ma and caused flattening of the slab^11^.

We can also observe in the Section A-A’ oriented along the subduction zone that the Benioff seismicity at the depth of 120–130 km forms a continuous lineament of earthquakes on both sides of the BN. This may indicate that the BN seismicity occurs in the middle part of the slab and is not associated with the interaction of different plates, as suggested by some authors^18,19^. The intensity of the BN seismic process is by more than an order of magnitude stronger than the background slab seismicity. The coincidence in space of the BN cluster with the slab-related seismicity shows that the subducting plate might be involved in the origin of this anomalous seismicity zone, which is the major difference distinguishing the BN from the cases of Vrancea^26^ and Pamir-Hindu Kush^25,29^.

**Figure 11:**
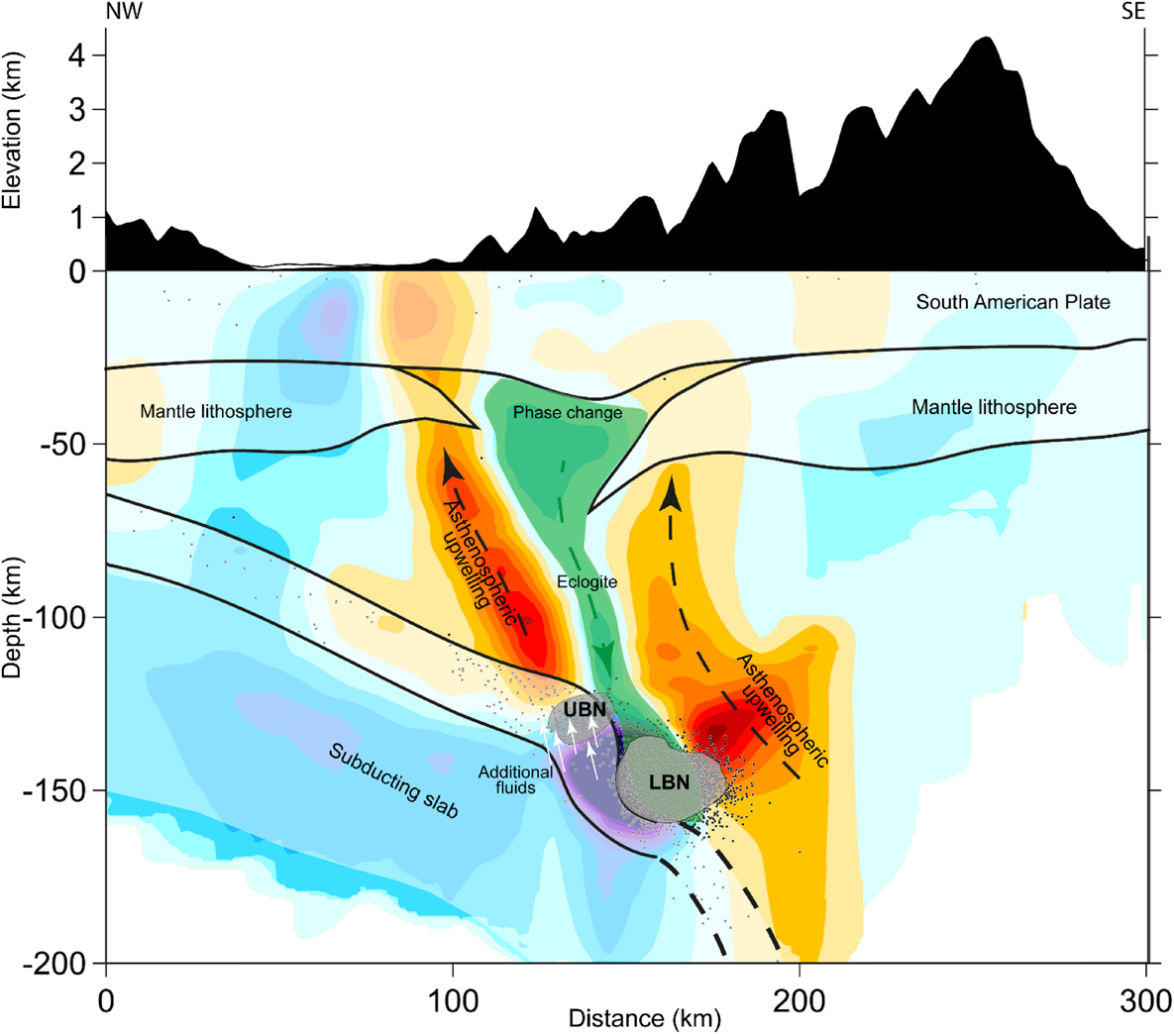
Geological interpretation based on seismic events and velocity anomalies. Background is the anomalies of Vp along section B-B’. Dots indicate seismic events at distances of < 20 km form the profile.

Our interpretation of the obtained velocity and seismicity distributions in the BN area is schematically presented in Fig. 11. We propose that the observed high-velocity anomaly within the BN represents the final stage of delamination, when a drip of higher density material was detached from the lithosphere and falling down to the subducting slab. The delamination scenario in similar settings was proposed by Kay and Kay^30^ and numerically simulated for the case of Central Andes Babeyko et al.^31^ and Sobolev et al.^32^. It has been shown that in the case of regional crustal shortening, some part of the lower crust may appear at large depths, which may cause transformation of mafic rocks to high-density eclogites. When reaching a certain critical mass, the eclogite body may cause gravitational instability and trigger the detachment of the mantle part of the lithosphere. As a result, the lithosphere and the lower-crustal material form the high-density drip, which is descending at a relatively high rate (up to 1 m per year^32^). This scenario was proposed for the cases of Vrancea^26^ and Pamir-Hindu Kush^25^. In NWSA, delamination has been proposed as mechanism related to the Paipa-Iza volcanic complex^33^, located north of Caldas tear and about 100 km south of the BN area. Some evidences for the lithosphere delamination in the Northern Volcanic Zone in Colombia were identified based on petrological studies of mantle and deep crustal xenoliths^34,35^.

We think that the same mechanism of delamination might be valid for the case of the BN. The distinct feature of the BN from other cases is that here the interaction of the falling drip with the rigid slab may affect the anomalous stress field in the contact zone that in turn may greatly intensify the seismic process. As we can observe in Fig. 11, the high-velocity anomaly at 130–160 km depth may represent the delaminated body entered to the slab. The exceptionally high values of seismic velocities might be explained by compaction of the material due to high pressure caused by the collision of the rapidly fallen delaminated body with the rigid slab. Above the high-velocity BN body, we observe a thin high-velocity anomaly of lower intensity that connects the BN with the bottom of the crust. It may represent a tail of the downgoing flow following the main delaminated drip, similarly as we can observe when we pour out a portion of viscous honey.

An important problem relates to the physical mechanisms responsible for intermediate-depth earthquakes. In the mantle at depths of more than 100 km, in the absence of fluid or melt, high lithostatic pressures make the usual dry frictional failure mechanism unlikely^36,37^. However, in subduction zones, some hydrated minerals, such as serpentine, undergo phase changes to anhydrous forms, releasing fluids in the process^2,38^. These fluids raise pore pressure, which in turn lower the effective pressure to values that permit brittle failure and earthquake occurrence^39,40^. As the subducting oceanic plate is rich in water, the intermediate-depth seismic activity connected with dehydration reaction is a common mechanism taking place in all subduction zones. For example, in our study we observe a clear belt of the regular Benioff seismicity at 130 km depth, which indicates zones of the dehydration reactions in the slab at this depth.

At the same time, it is very rare when the intermediate-depth seismicity occurs below continents. Although, the continental collision is often associated with descent of large lithosphere masses, in most cases it is not associated with mantle seismicity. In normal conditions, the lower crust and mantle lithosphere that deepen due to collisional processes do not contain sufficient amounts of water and therefore cannot initiate the seismogenic process. For the exceptional cases of Vrancea or Hindu Kush, the seismicity might be associated with the presence of remnant oceanic lithosphere. In both cases, the geological history shows that the phase of continental collision was preceded by closure of paleo oceanic basins, hence the hypothesis on sinking the remnant oceanic lithosphere in these areas is plausible.

For the area of the BN, the situation is completely different, as it is located inside a well-developed continent, where there was no trace of any relict oceanic basins. One of the mechanisms of seismicity generation in the mantle could be thermal shear instability, as was proposed for the BN^2,27,41,42^, as well as for the Hindu-Kush nest^43^.

As an alternative scenario, we propose that the main cause of such a strong seismicity in the BN is the interaction of the “dry” delaminated drip with “wet” subducting slab. The strong impact of the rapidly fallen delaminated body with the slab may intensify the processes of dehydration. One possible mechanism of intensification could be the fact that the delaminated body entered inside the subducting plate and triggered dehydration reactions in layers located inside the slab, which produced much more water than during a regular subduction process, when mostly near-surface layers are involved.

The differentiation between two distinct seismicity clusters called UBN and LBN, as presented in Fig. 11, allows us to reconcile two mechanisms responsible for intermediate-depth seismicity, fluid-induced embrittlement and delamination. The former has been proposed for the BN by several authors^2,3,27,44^, and considered as the main mechanism to trigger seismicity at mid-depth^45^. We interpret dehydration embrittlement as the seismic activity of the clustering observed at 130 km, which includes the UBN. As for the LBN, given that the *Vs* values are high, thus implying a low content of fluids, we can rule out dehydration inside the delaminated body; instead, we propose intensified dehydration in the slab as the driving mechanism of seismicity.

## Conclusions

In this study, we reprocessed the regional catalog data provided by National Seismological Network of Colombia (NSNC), which was previously used in a number of tomography studies of the Colombian region. Here, we mostly focused on studying the structure of the BN and paid much attention on assessing the resolution limitations of the results. As a result of the tomographic inversion, we have obtained a prominent anomaly of high *Vp* and high *Vs* coinciding with the distribution of the intermediate depth seismicity.

We propose that this anomaly represents the delaminated material (eclogitic drip with high density and low porosity and permeability) that was detached from the lower part of the continental lithosphere and fallen down to the slab. We propose that the impact of this rapidly descending delaminated block with the subducting plate has intensified the dehydration processes inside the slab. The release of anomalous amounts of water and high stresses associated with the impact are the main causes of the exceptionally strong seismicity observed within the BN.

Such interactions of delaminated bodies with the slab should be a common scenario that may take place when subduction occurs below a continental type of the lithosphere. For example, such a mechanism was considered in numerical simulations for the Central Andes settings^32^. Similar interaction of the delaminated material with the slab, but at a later stage, was identified below Hokkaido based on the results of seismic tomography^24^. The reason why the nests like Bucaramanga are not observed in other places, is that on a geological time scale, the occurrence of such an impact process should be very fast. As the time of its active phase is much shorter than the periods of accumulation and falling of the delaminated material, it is unlikely that such impacts occur simultaneously in several places. Hence, Bucaramanga is a unique location, where this process causing a strong seismic activity occurs nowadays.

## Methods

### General workflow of the LOTOS code for the local earthquake tomography

A 3D velocity tomography inversion was performed using the LOTOS code^9^. The input data for the calculations are the seismological station coordinates and arrival times of the P and S waves from local events, as well as a number of controlling parameters and a starting velocity model. For tomography, we selected data using three criteria: (1) the distance to the nearest station should be smaller that 200 km; (2) the number of the P and S phases per event should be equal or larger than 17; and (3) the residuals after the step of preliminary location should be smaller than 1.5 s.

We used the 1D starting velocity model with a constant Vp/Vs ratio equal to 1.75. The P-wave velocities were defined at several depth levels, as presented in Table 1, and were linearly interpolated. This model was previously used for another subduction zone in Kamchatka^46^. Note however that for dominantly steep rays from deep sources, the residuals are not strongly dependent on the absolute velocities in the reference model. Therefore, similar shapes of anomalies were obtained when considerably different starting velocities were used.

**Table 1:** P-wave velocity distribution in the 1D starting velocity model.

Depth, km	P-wave velocity, km/s
− 5	6.0
15	6.7
40	7.8
120	8.0
165	8.1
210	8.2

The initial source locations in the starting 1D velocity model are calculated with the use of the grid-search method, which enables stable source coordinates even if the initial search point is located far from the true location. The travel times at this step are calculated using previously calculated tabulated values. In the next step, the sources are located using the gradient descent method and more sophisticated algorithm of 3D ray tracing based on the bending method according to the Fermat’s Principle. This step is repeated iteratively after each update of the 3D velocity model.

The 3D velocity model was parameterized using a set of nodes distributed in the study area according to the data distribution. In map view, the nodes are distributed regularly with the spacing of 15 × 15 km merely in areas with sufficient ray coverage. In the vertical direction, the spacing between nodes was inversely proportional to the ray density, but could not be smaller than 10 km. To minimize the dependency of the results on the grid geometry, we performed several inversions in grids with different basic azimuthal orientations (0, 22, 45 and 64 degrees) and then stacked results.

The inversion was performed simultaneously for the 3D P and S wave velocity anomalies and source parameters (coordinates and origin times) using the LSQR method^47,48^. The stability of the inversion was controlled by additional matrix blocks responsible for the amplitude damping and flattening of the resulting velocity anomalies. The coefficients for the inversion (amplitude damping of 7 and 12 and flattening of 3 and 6 for dVp and dVs, respectively) were defined from synthetic modeling as providing the best recovery of the known structures.

The starting velocity distribution was assessed using a series of full tomography runs with iterative changes of the starting velocities according to the results of the previous iterations to enable an optimal balance between the positive and negative anomalies in the resulting model.

The inversion results are presented as distributions of the P and S wave velocity anomalies in two horizontal and two vertical sections in the main paper in Figs. 4 and 5. Note however that the Vp/Vs ratio was not stable in this particular case as it was strongly dependent on the balance between the damping values for the P and S anomalies. That is why this parameter is not used for the interpretation.

To assess the reliability of the derived 3D seismic velocity structures in the BN area, we performed a series of synthetic tests that simulated the conditions of the experimental data inversion. To produce the synthetic dataset, we used the same event locations and same source-receiver pairs as obtained in the final solution of the main model. The synthetic travel times were calculated by the bending ray-tracer in a predefined 3D synthetic velocity model. Then the travel times were perturbed with random noise having the mean deviation of 0.1 s and 0.2 s for the P and S wave data. Before starting the recovery of the model, the locations and the origin times of sources in the synthetic dataset were “forgotten”. The recovery procedure was identical to the case of experimental data inversion including the step of initial source location. The controlling parameters for the inversion were also identical.

## Numerical modeling

To simulate the scenario of the BN development (Fig. 10), we used hydromechanical subduction model^28^ allowing to compute material deformation and fluid motion in a (de)compacting viscous matrix filled with porous aqueous fluid. Governing conservation equations are solved with staggered finite differences combined with marker in cell method for representation of material properties. The following governing equations are solved^28^:

Mass conservation for solid matrix,

$$div\left({\overrightarrow{v}}^{s}\right)+\frac{{P}^{t}-{P}^{f}}{(1-\phi ){\eta }^{\phi }}=0,$$

Mass conservation for aqueous fluid,

$$div\left({\overrightarrow{q}}^{D}\right)-\frac{{P}^{t}-{P}^{f}}{\left(1-\phi \right){\eta }^{\phi }}=0.$$

Momentum conservation for bulk material (solid + fluid) (Stokes equation),

$$\frac{\partial {\tau }_{ij}}{\partial {x}_{j}}-\frac{{\partial P}^{t}}{\partial {x}_{i}}+{\rho }^{t}{g}_{i}=0,$$

Momentum conservation for fluid (Darcy equation),

$${q}_{i}^{D}=-\frac{k}{{\eta }^{f}}\left(\frac{{\partial P}^{f}}{\partial {x}_{i}}+{\rho }^{f}{g}_{i}\right)=0,$$

where

$${\tau }_{ij}=2{\eta }^{t}{\dot{\varepsilon }}_{ij}^{{\prime}s},$$

$${\dot{\varepsilon }}_{ij}^{{\prime}s}=\frac{1}{2}\left(\frac{\partial {v}_{i}^{s}}{\partial {x}_{j}}+\frac{\partial {v}_{j}^{s}}{\partial {x}_{i}}\right)-{\delta }_{ij}\frac{1}{2}div\left({\overrightarrow{v}}^{s}\right),$$

$${\eta }^{t}={\eta }^{s}{e}^{-28\phi },$$

$${\eta }^{\phi }=\frac{{\eta }^{s}}{\phi },$$

$$k={k}_{0}{\left(\frac{\phi }{{\phi }_{0}}\right)}^{3}{\left(\frac{{1-\phi }_{0}}{1-\phi }\right)}^{2},$$

$${\rho }^{t}={\rho }^{f}\phi +{\rho }^{s}\left(1-\phi \right),$$

$${P}^{t}={P}^{f}\phi +{P}^{s}\left(1-\phi \right),$$

where ${\tau }_{ij}$ is deviatoric stress, ${\dot{\varepsilon }}_{ij}^{\mathrm{^{\prime}}s}$ is deviatoric strain rate, ${\overrightarrow{v}}^{s}$ is solid matrix velocity, *P*^*t*^ is total pressure, *P*^*f*^ is fluid pressure, *P*^*s*^ is pressure of the solid, $\phi$ is porosity (volumetric fluid fraction), ${\eta }^{t}$ is the effective shear viscosity of the bulk material, ${\eta }^{\phi }$ is compaction viscosity, ${\eta }^{s}$ is viscosity of the solid, ${\eta }^{f}=$ 10^–3^ Pa.s is aqueous fluid viscosity, *k* is permeability of the matrix, *k*_0_ is permeability at standard porosity ${\phi }_{0}=0.01$, ${\overrightarrow{q}}^{D}$ is Darcy flux, ${\rho }^{t}$ is density of the bulk material, ${\rho }^{s}$ is density of the solid, ${\rho }^{f}$ is fluid density (1000 kg/m^3^), ${g}_{i}$ is gravity acceleration (purely vertical, 10 m/s^2^), ${\delta }_{ij}$ is Kronecker delta.

Numerical model geometry is shown in Fig. 9 and consists of 690 × 390 km computational domain resolved with 346 × 196 regular staggered grid and 1,076,400 randomly distributed Lagrangian markers. The model setup corresponds to 70 km thick slab subducting into the mantle with 5 cm/yr velocity. The mantle wedge atop the slab is hydrated and contains dense circular object (eclogitic drip) with lowered porosity and respectively lowered permeability. Both the mantle and the slab are anhydrous. Low-viscosity, low-density sticky water layer is present on top of the model to simulate free surface boundary conditions. Material properties are as follows: sticky water ($\phi ={10}^{-4}$, *k*_0_ = 10^–17^ m^2^, ${\rho }^{s}$=1000 kg/m^3^, ${\eta }^{s}$ = 10^17^ Pa.s), dry mantle ($\phi ={10}^{-4}$, *k*_0_ = 10^–17^ m^2^, ${\rho }^{s}$=3300 kg/m^3^, ${\eta }^{s}$ = 10^20^ Pa.s), wet mantle wedge ($\phi =0.01$, *k*_0_ = 10–^17^ m^2^, ${\rho }^{s}$=3300 kg/m^3^, ${\eta }^{s}$ = 10^19^ Pa.s), slab ($\phi ={10}^{-4}$, *k*_0_ = 10^–17^ m^2^, ${\rho }^{s}$=3330 kg/m^3^, ${\eta }^{s}$ = 10^23^ Pa.s), eclogitic drip ($\phi =0.005$, *k*_0_ = 10^–17^ m^2^, ${\rho }^{s}$=3500 kg/m^3^, ${\eta }^{s}$ = 10^18^ Pa.s).

Boundary conditions correspond to the constant 4.73 cm/yr influx velocity at the left boundary compensated by the 2.68 cm/yr outflux velocity at the lower boundary to ensure volume conservation. Right and top boundaries are free slip. All boundaries are insulated for fluid flux. Calculations are performed for single time step to obtain solution for *P*^*t*^, *P*^*f*^, ${\overrightarrow{v}}^{s}$ and ${\overrightarrow{q}}^{D}$. Brittle-plastic strength of the fluid-bearing mantle (Fig. 10c) is computed as

$${\sigma }_{yield}={\sigma }_{c}+\mu \left({P}^{t}-{P}^{f}\right),$$

where ${\sigma }_{c}$= 4 MPa is compressive strength and $\mu$=0.6 is internal friction coefficient.

## Data Availability

The results of this study can be reproduced using the data and the LOTOS codes files openly available in Zenodo at: Perez-Forero, D. (2023). LOTOS code for the local earthquake tomography in the Bucaramanga nest, Colombia. https://doi.org/10.5281/zenodo.7947754.
